# Determination of the effects of different high‐temperature treatments on texture and aroma characteristics in Alaska pollock surimi

**DOI:** 10.1002/fsn3.763

**Published:** 2018-10-10

**Authors:** Hua Zhang, Yaozhou Zhu, Shi Chen, Changhua Xu, Yan Yu, Xichang Wang, Wenzheng Shi

**Affiliations:** ^1^ College of Food Science and Technology Shanghai Ocean University Shanghai Engineering Research Center of Aquatic Product Processing and Preservation Shanghai China; ^2^ Department of Food Science and Human Nutrition University of Florida Gainesville Florida

**Keywords:** Alaska pollock surimi gel, gel properties, high‐temperature treatment, quality, volatile component

## Abstract

This study tested the gel properties, quality of Alaska pollock surimi subjected to different temperature treatments. Results showed that when the heating temperature is 110°C, the water‐holding capacity (WHC) and texture of the surimi and gel strength increased, but as the heating temperature increased, the gel strength decreased significantly (*p* < 0.05), and ultimately destroyed. The heating temperature had no significant effect on the whiteness of the surimi gel, although it did have a significant effect on volatile components (*p* < 0.05). Fourier transform infrared (FT‐IR) spectroscopy suggested that with increasing temperature, protein secondary structure of the random coil received maximum damage, leading to protein aggregation and ultimately greatly reduced gel strength. At 100, 105, 110, 115, and 121°C, the surimi gel was determined 37, 46, 49, 52, and 56 volatile components, from of aldehydes, ketones, alcohols, hydrocarbons, and aromatic compounds. These results indicate that heat treatments have an important influence on the gel properties and volatile components of Alaskan pollock surimi gel, and the treatment parameters can be valuable for the production of ready‐to‐use minced fish products.

## 1 INTRODUCTION

Surimi products (surimi comprising mainly salt‐soluble myofibril proteins) are a rich source of nourishment. They are consumed not only as snacks, but also as hotpot ingredients (Jafarpour & Gorczyca, 2008). Surimi products can be added to improve the flavor and nutrition of numerous dishes. In fact, surimi is a basic ingredient in alternative seafood products and is valued for its unique gelling properties. Demand for surimi products continues to rise as consumers in both developed and developing countries pay more attention to healthy foods rich in proteins and low in fat (Muriel‐Galet, López‐Carballo, Gavara, & Hernández‐Muñoz, 2014).

There are many ways to study volatile substances. The accurate extraction of volatiles is critical in this process, and various extraction methods are used for volatile organic compounds for the whole determination (VOCs) in seafood, such as steam distillation (SD) “purge and trap” (P&T) (Conde‐Hernández, Espinosa‐Victoria, Trejo, & Guerrero‐Beltrán, 2017), dynamic headspace (DHS), simultaneous distillation extraction (SDE) (De Frutos, Sanz, & Martínez‐Castro, 1988; Varlet, Prost, & Serot, 2007), vacuum distillation (VD) (Pennarun & Prost, 2002), and solid‐phase microextraction (SPME) (Oh & Shin, 2017). Solid‐phase microextraction (SPME) is a fast, sensitive, and economical method for sample extraction before gas chromatography analysis in comparison with other well‐established techniques for analyzing volatiles in food. The technique was developed by Arthur and coworker (Arthur & Pawliszyn, 1990) and combines one‐step sampling and sample preparation. In recent years, it has been successfully used for determining the flavor profiles of several fish species (Igiesias, Gaiiardo, & Medina, 2010). In addition, the e‐nose is a useful instrument for analyzing, identifying, and detecting complex smells. Due to its proven characteristics of good reproducibility, undamaged samples, and shortcuts, it provides an effective reference for the aroma and quality of aquatic products. This study, therefore, solid‐phase microextraction gc‐ms and e‐nose, was used to investigate the influence of high‐temperature treatment on the volatile components of surimi.

Many previous studies have focused on the effects of heat treatment on gel and volatile organic compounds (VOCs) in surimi products. However, in previous work, the surimi processing temperatures were lower than 100°C, and the samples were stored at −18°C. Few have reported on the effects of temperatures above 100°C. The surimi products’ whiteness, water‐holding capacity, volatile compounds, and protein secondary structure were studied. The study aims to obtain the optimal heat treatment temperature of surimi products. The findings could provide important theoretical and practical guidance for the surimi products.

## 2 MATERIAL AND METHODS

### 2.1 Samples gel preparation

Frozen surimi (300 g) was tempered at 4°C for 4 hr before being cut into small pieces (3 cm cubes). The surimi cubes were placed in a cutter (Model UM5) and chopped at a low speed (500 g) for 5 min. Over the following 3 min, NaCl (7.5 g) was sprinkled into the surimi, during which time the mixture was chopped at speed of 700 g. The entire chopping process was carried out below 10°C. Finally, the surimi was poured into the plastic casing to make fish sausages, before being heated in a two‐temperature‐process: first smoldering and then high‐temperature treatment at 100, 105, 110, 115, and 121°C, for 10 min. After sterilization, the surimi was packaged and stored at −4°C for further determination.

### 2.2 Determination of WHC

Using the method published by Ng (1987), the WHC was then calculated as follows:

$$\text{WHC}=[1-\left(X_{1}-X_{2}\right)/X_{1}]\times 100\%$$

### 2.3 Measurement of textural properties

Texture profile analysis (TPA) is important indicators of quality, including hardness, elasticity, cohesion, and mastication. TPA and the gel strength were estimated by a TA‐XT plus texture analyzer according to the procedure described in Lanier (1992).

### 2.4 Determination of whiteness

The color of the surimi gels was determined by measuring the L*(lightness), a*(redness/greenness), and b* (yellowness/blueness) values. The whiteness was calculated using the following equation:

$$\text{Whiteness}=100-{\left[{\left(100-L*\right)}^{2}+a^{*2}+b^{*2}\right]}^{1/2}$$

### 2.5 Gel strength analysis

The gel strength was analyzed using the Texture Profile Analyzer (TMS‐PRO, Food Technology Corporation, Virginia, USA) according to Qu's method (Qu et al., 2012). The gel was subjected to a compression test with a trigger‐type button. A cylindrical probe with a 5‐mm diameter was used to penetrate the sample at 20 mm. The trigger force was 0.5 N, with a test speed of 60 mm/min. The sample deformation was 60%, and the gel strength (g × mm) was equal to the breaking strength (g) multiplied by the breaking distance (mm).

### 2.6 FT‐IR spectroscopy analysis

Fourier transform infrared (FT‐IR) spectroscopy of samples was carried out in a 470 FT‐IR spectrometer (Thermo Fisher Instruments Co., Ltd., Shanghai, China). The samples were freeze‐dried and then mixed with pure KBr. The mixture was subsequently ground to uniformity, placed in an agate mold, and then pressed into a diaphanous sheet with a (5–10) × 10^7 ^Pa pressure on the hydraulic machine. The measurement conditions were as follows: the range of wave number was 4,000–400 cm^−1^, the resolution was 4 cm^−1^, and the number of spectra collected was 32.

The FT‐IR of the samples was processed and fitted by OMNIC and Peak Fit software. First, baseline correction was conducted for the amide I band (1,600–1,700 cm^−1^) of samples, and then 9‐point Savitzky–Golay function smooth, Fourier deconvolution, and second derivative were processed. The Gaussian curve fitting was performed with the Peak Fit so that the overlapped bands completely separated, and obtained the minimized residuals.

### 2.7 E‐nose analysis of volatile compounds

The surimi gel samples were further analyzed by a FOX‐4000 sensor array system (Alpha M.O.S., France) to distinguish discrepancies between the aroma profiles of various fish samples rinsed by different high‐temperature treatments. This instrument consists of a detector unit, auto‐sampling device, and pattern recognition software for data recording and interpretation (Wang, Wang, Liu, & Liu, 2012). Five grams of sample was loaded into a 10‐ml glass vessel and placed on a specimen tray (4°C) for detection. For each sample, the e‐nose detection was repeated eight times under the same conditions.

### 2.8 GC–MS settings

GC–MS analysis was performed in a Thermo Finnigan Thermo Quest (San Jose, CA, USA) gas chromatograph, equipped with a split/splitless injector and coupled with a trace quadrupole mass detector. Compounds were separated in a capillary column (60 m × 0.32 mm × 1 μm film thickness, DB‐5MS). The temperature program was as follows: initial temperature of 40°C held for 3 min; from 40 to 100°C at 5°C/min, from 100 to 160°C at 5°C/min, from 160 to 250°C at 12°C/min, and this final temperature was held for 3 min. Helium was employed as carrier gas, with a constant flow of 1.0 ml/min. The injector was operated in the split mode with temperature set at 260°C. Transfer line temperature was maintained at 280°C. The quadrupole mass spectrometer was operated in the electron impact (EI) mode with the source temperature set at 230°C. Initially, full scan mode data were acquired to determine the appropriate masses for later acquisition in selected ion monitoring mode (SIM), under the following conditions: a mass range of 35–350 amu and a scan rate of 0.220 s/scan. All analyses were performed with ionization energy set at 70 eV, filament emission current set at 150 μA, and the electron multiplier voltage set at 500 V.

### 2.9 Qualitative and quantitative analysis of volatile compounds

The volatile compounds analyzed by GC–MS were identified by matching the mass spectra obtained to those gathered in the NIST 02 library and the Wiley database. Only compounds in which the positive or negative matching degrees were greater than 800 (maximum is 1,000) were reported (Diao et al., 2016; Shi, Chen, You, & Wang, 2014). In addition, 10 μl of hydrocarbon mixture of C7–C30 (as an internal standard) was added to 5 g of homogenized sample immediately before the headspace SPME process.

The relative content of each component in the volatile components of the different samples was obtained using the area normalization method, and the experiment results were compared with the least significant difference method (Wang & Yang, 2010).

### 2.10 Statistical analysis

Analyses of variance (ANOVA), multiple comparisons by Tukey's HSD method, and correlation analysis were carried out using SPSS software (version 17, IBM Institute Inc.). All of the data were represented as mean ± *SD*.

## 3 RESULTS AND DISCUSSION

### 3.1 Effect of high‐temperature treatment on the WHC of surimi gel

Water‐holding capacity is a crucial physical parameter closely related to the quality of surimi gels. The higher the WHC values, the less the internal water is pressured, and it indicated the surimi gel maintains more water.

**Figure 1:**
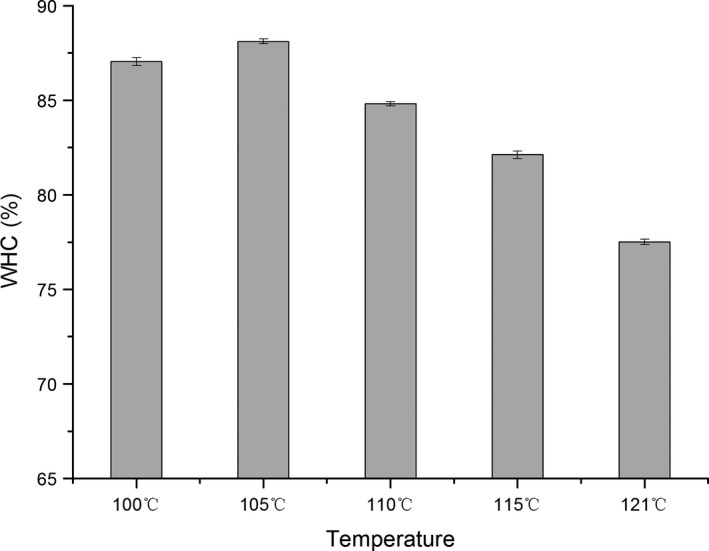
WHC of high‐temperature treatment surimi gels. WHC, water‐holding capacity

The WHC values for surimi gels carried at different temperatures from 90 to 120°C are shown in Figure 1. WHC decreases as the temperature rise. Although there was no significant change at 100–115°C, during which the surimi gel maintained a balanced stage as the temperature increased from 115 to 121°C, the quality of the surimi gel declined sharply, thus suggesting severely disrupted gel network structure, leading to a serious decline in WHC. A high quality of surimi gel is reflected in the density of the gelatinous structure. A uniform dense gel is beneficial to the retention of moisture in the gel network, thus, increasing the WHC of the surimi gel.

The significance of WHC can be further explained as follows: The water structure and form of the gel system is varied. As the fish protein and water H‐bound, ionic bond and hydrophobic interaction in the gel, creating crosslinking gaps, so the moisture will be locked in certain areas, improving the water retention of the gel system. The entire network structure is thus more closely knit, thereby, improving the gel characteristics (Sánchez‐González et al., 2008). High‐temperature treatments may undermine the gel properties and change the three‐dimensional network structure, thus decreasing the gel's WHC. Thus, WHC decreased as the heating temperature increased from 100 to 121°C for all the samples.

### 3.2 Effects of different high‐temperature treatments on the textural properties (TPA) of surimi gel

Major surimi gel quality texture parameters, including hardness, elasticity, chewiness, and cohesiveness, were analyzed using TPA (texture profile analysis). The sample was compressed twice in a simulated chewing movement of the human oral cavity, and thus providing a texture test curve to understand sensory evaluation‐related quality and structure parameters.

Heat treatment was found to have an effect on the quality and structure of surimi gel and is shown in Table 1. As the temperature increased from 100 to 121°C, the gel's hardness, elasticity, chewiness, and cohesiveness all have different degree of reduction. High‐temperature treatments reduced the hardness of fish sausage significantly (*p* < 0.05), in 115 and 121°C, although there was no significant difference in hardness between 105 and 110°C (*p* > 0.05). High‐temperature treatment had insignificant influence on elasticity and cohesion. Chewiness is a comprehensive evaluation of the texture and is numerically expressed as hardness, cohesion, and elasticity (Hu, 2010). The chewiness of the surimi sausage samples decreased significantly at 121°C, but not significantly (*p* > 0.05) altered at 100 and 105°C. Overall, from the perspective of TPA, the most suitable temperature for high‐temperature treatment was determined to be 110°C. In previous studies, many articles reported that the higher the temperature, the greater the impact on the texture profile analysis (TPA) of surimi gel.

**Table 1:** Effects of high‐temperature treatment on TPA parameters of surimi gels

	100°C	105°C	110°C	115°C	121°C
Hardness/g	4993.15 ± 149.36d	4211.02 ± 206.05c	4046.45 ± 144.64c	3642.82 ± 183.25b	3252.44 ± 146.91a
Springiness	0.88 ± 0.01ab	0.89 ± 0.02b	0.87 ± 0.04ab	0.86 ± 0.02ab	0.84 ± 0.01a
Cohesiveness	0.88 ± 0.04c	0.82 ± 0.06bc	0.84 ± 0.09c	0.74 ± 0.04b	0.62 ± 0.03a
Chewiness/g	4123.86 ± 218.16d	3119.59 ± 207.71c	2461.49 ± 152.58b	2170.32 ± 187.53ab	2022.45 ± 117.69a

The mean values marked with lowercase letters in ascending order show significant statistical differences within rows (p < 0.05).

### 3.3 Effect of different high‐temperature treatments on the whiteness of surimi gel

Whiteness is a significant quality indicator for surimi products (Park, 1995). Generally speaking, consumers have the highest demand for surimi gel with high L* value, low b* value, and high whiteness (Hsu & Chiang, 2002).

**Figure 2:**
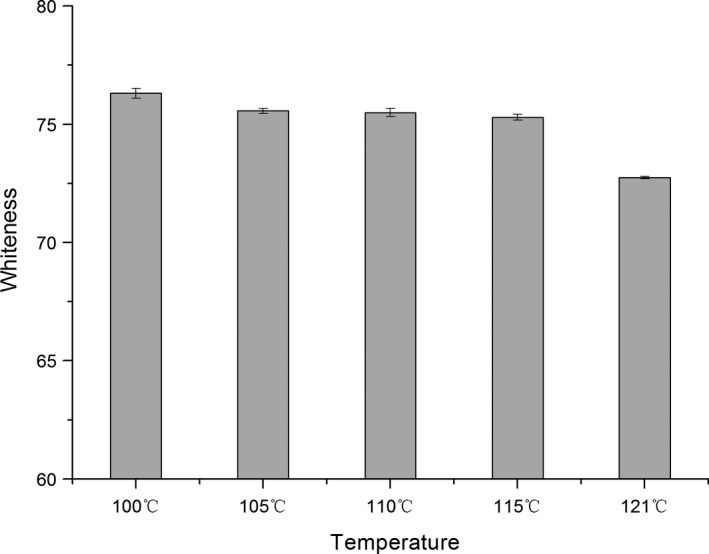
Whiteness of high‐temperature treatment surimi gels

Figure 2 shows the effect of different high‐temperature treatments on the whiteness of surimi gel samples heated from 100 to 121°C. When the heating temperature increased from 100 to 121°C, the surimi gel showed a trend of slow decline in its whiteness, though the change is not statistically significant. In the entire process of heat treatment, the surimi gel maintained its white color, with no obvious change, however, when the temperature up to 121°C, the color changed from white to pale yellow. This difference may be explained by the rearrangement of water molecules during the formation of surimi gel. After high‐temperature treatment, the position of the water molecules is disturbed, leading to their rearrangement, which probably affects the transparency and color of the surimi gels. These results indicated the change in surimi whiteness during heat treatment is within an acceptable range. This will be of great significance for ready‐to‐eat surimi products. High‐temperature treatments have been found not only destroyed the noncovalent bonds, but also to affect or destroy the covalent bond formation or destruction, thus further affecting the extent and nature of protein denaturation, and changing the whiteness of the surimi gels (Zhang, Xue, Xu, Li, & Xue, 2013). In previous studies, canned fish balls sterilized at 116°C for 30 min were found to have a significantly lower whiteness in the surimi gel (Runglerdkriangkril, Banlue, & Raksakulthai, 2008). This finding is consistent with a study by Park (1995). As shown in Figure 2, in this study, the gel whiteness decreased as the treatment temperature increased from 100 to 121°C.

### 3.4 Gel strength analysis

**Figure 3:**
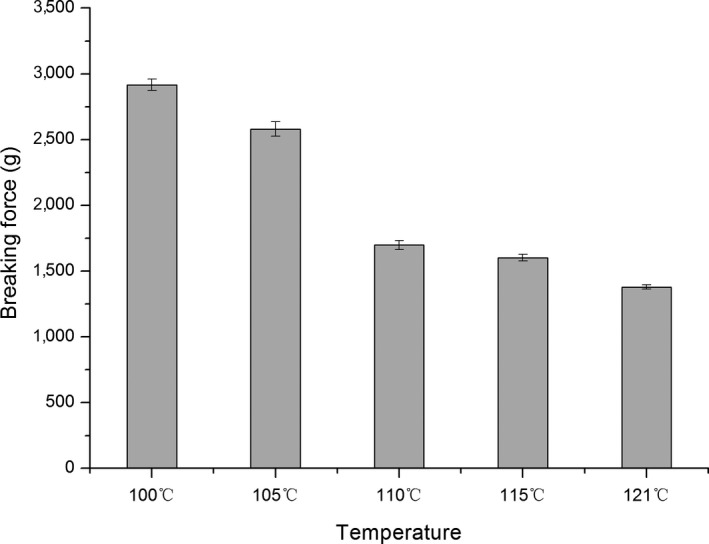
Effects of high‐temperature treatment on the breaking force of surimi

**Figure 4:**
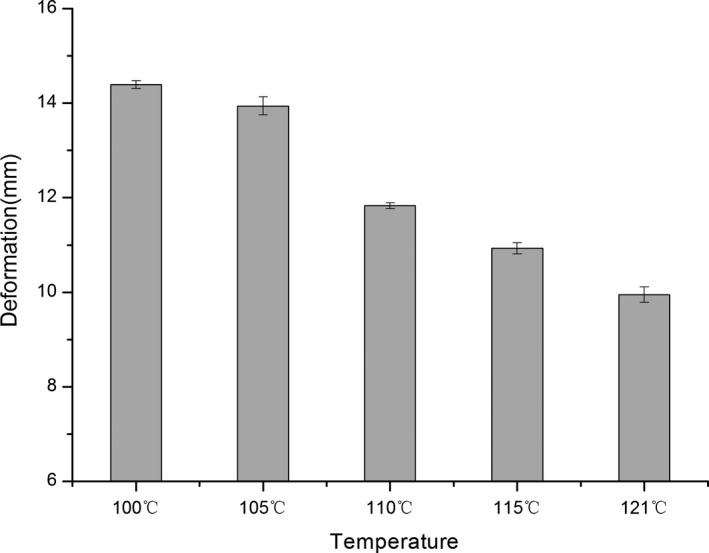
Effects of high‐temperature treatment on the deformation of surimi

**Figure 5:**
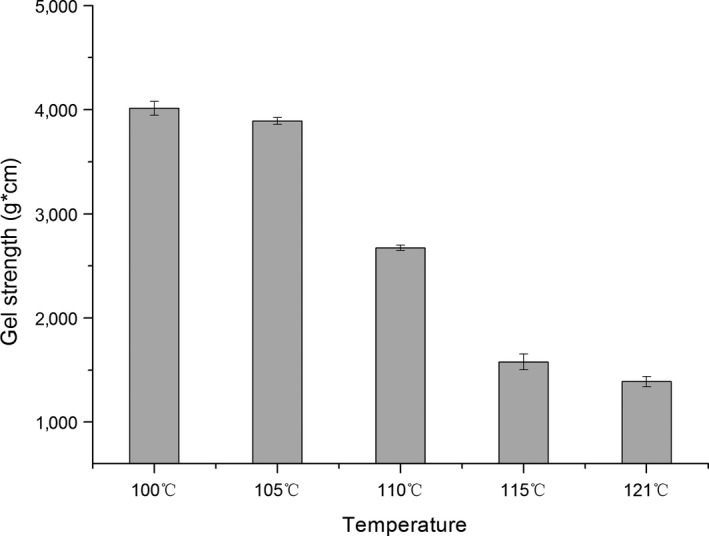
Effects of high‐temperature treatment on the gel strength of surimi

Significant changes in the surimi gel strength were induced by high‐temperature treatment as presented in Figures 3, 4, 5. The breaking strength of the surimi gel was found to be 100–121°C with sag distance increasing and gel strength decreased to some degree. When the temperature rose to 110°C, the gel strength had been completely destroyed. Whereas the temperature continues to rise, the gel strength drops sharply, reaching a minimum of 1,383.87 g × cm. In addition, the gel strength was a maximum 100°C, which was 4,069 ± 490 g × cm. This is consistent with a previous study showing that high‐temperature treatment could lead to the change in the quality of fish. In Shie and Park (1999)'s study, when the fish sausage was heated to 121°C for 30 min, the breaking strength and the degree of sag dropped significantly reported the deterioration of gel strength under three temperatures (75, 85, and 93°C) for 0–120 min, while samples processed at temperature below 100°C produced different results.

The main protein in surimi gels is myofibril, which mainly includes actin and myosin. Protein is easily denatured under high‐temperature conditions. The heat resistance of surimi in high‐temperature treatment was therefore studied. The gel strength properties of Spanish mackerel's surimi in canned fish balls were determined to be reduced by heat treatment conditions of 116°C for 30 min (Runglerdkriangkrai, Banlue, Raksakthai, 2008).

### 3.5 FT‐IR spectroscopy

The protein secondary structure refers to the spatial structure of the local peptide segments in the polypeptide chain skeleton, without considering the side chain conformation and the spatial arrangement of the integrated peptide chains, which largely determine the functional properties of protein (Lu, Zhang, & Wang, 2008). The forms of α‐helix, β‐fold, β‐corner, and random coil, among others, constitute the essential secondary structural elements of the high protein structure. FT‐IR spectroscopy was the earliest and most common way to study protein conformation and was thus applied in this study (Argyri et al., 2013).

**Figure 6:**
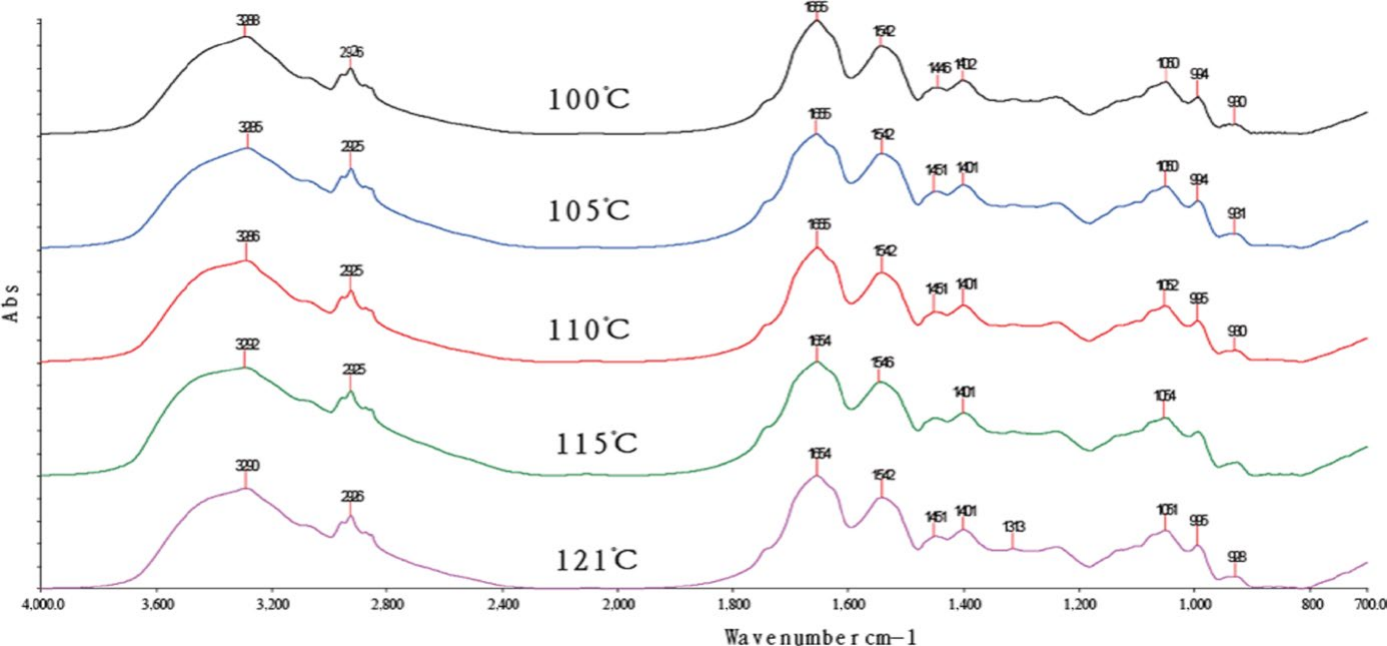
FT‐IR spectra of the surimi gels with different high‐temperature treatment. FT‐IR, Fourier transform infrared

**Figure 7:**
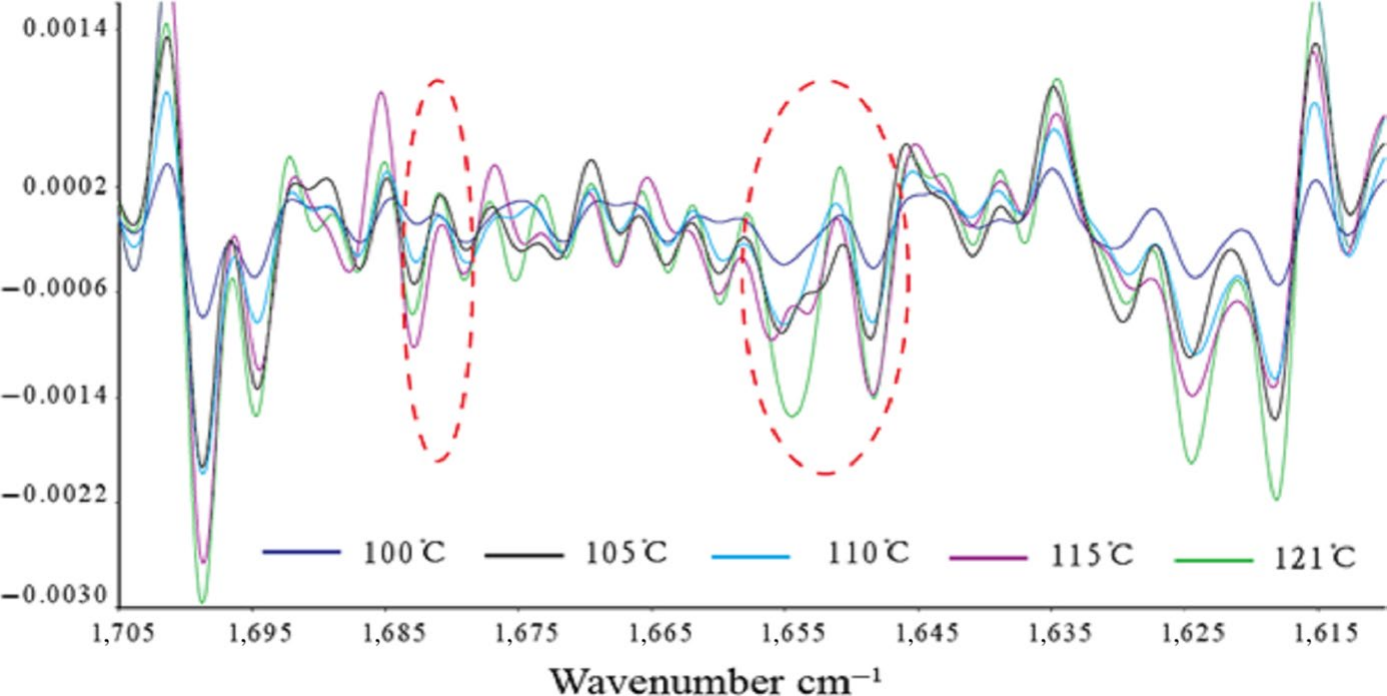
Second derivative spectra of surimi gel in the region of 1,705–1,615 cm^−1^

The absorption of the characteristically strong peak of amide I bands is often used to analyze the secondary structure of proteins. The relationship between the protein secondary structure and the peaks is as follows: 1,650–1,658 cm^−1^ for the α‐helix, 1,610–1,640 cm^−1^for β‐fold, 1,660–1,695 cm^−1^ for β‐corner, and 1,640–1,650 cm^−1^ for the random coil (Liu et al., 2015). As shown in Figures 6, 7, the characteristic peak in this study appeared in the vicinity of 1,645 cm^−1^. The peak was judged as β‐fold and random coil, indicating that the protein secondary structure of the surimi gels treated with high temperature was mainly a random coil and β‐fold.

**Figure 8:**
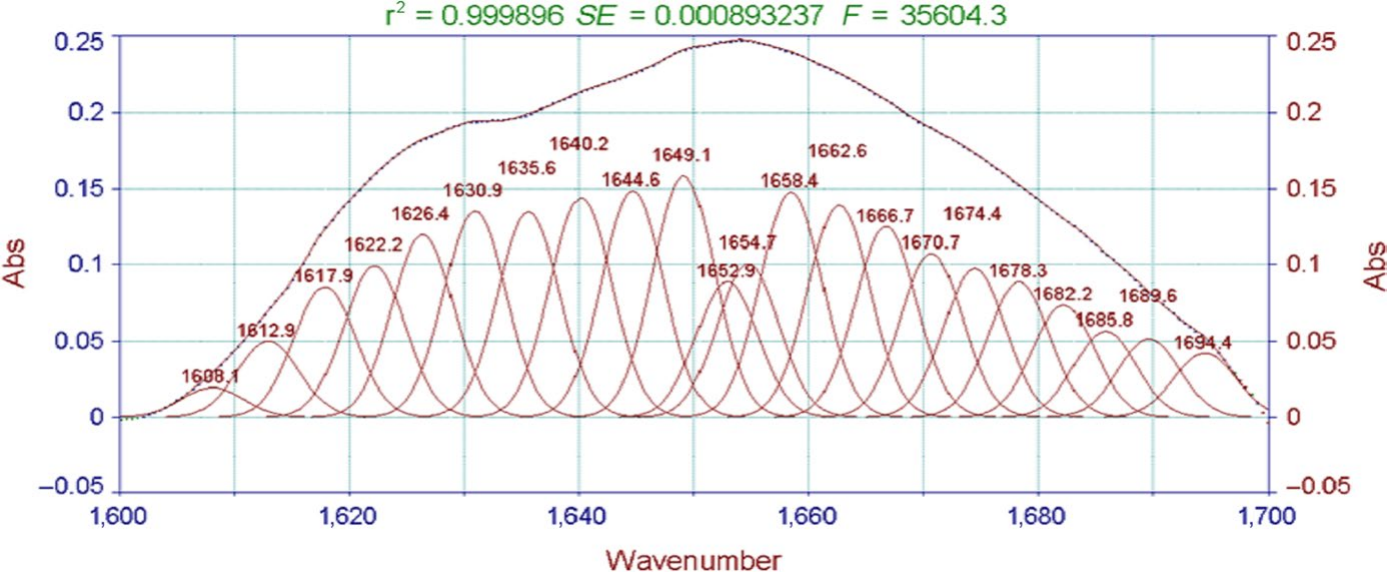
The fitting figures of amide I in surimi gel at 100°C

**Figure 9:**
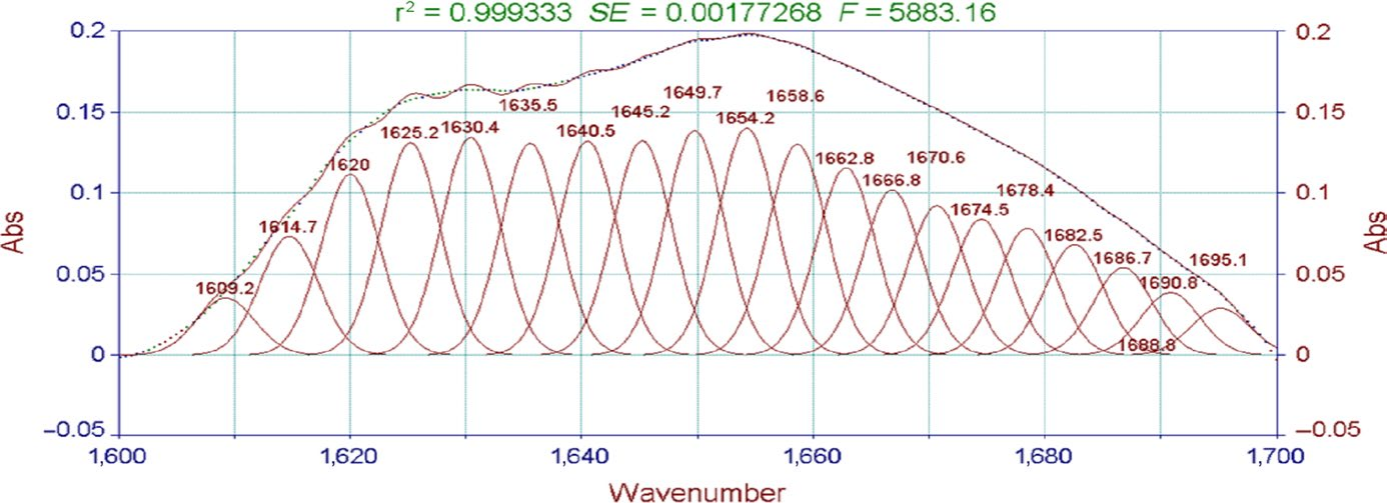
The fitting figures of amide I in surimi gel at 105°C

**Figure 10:**
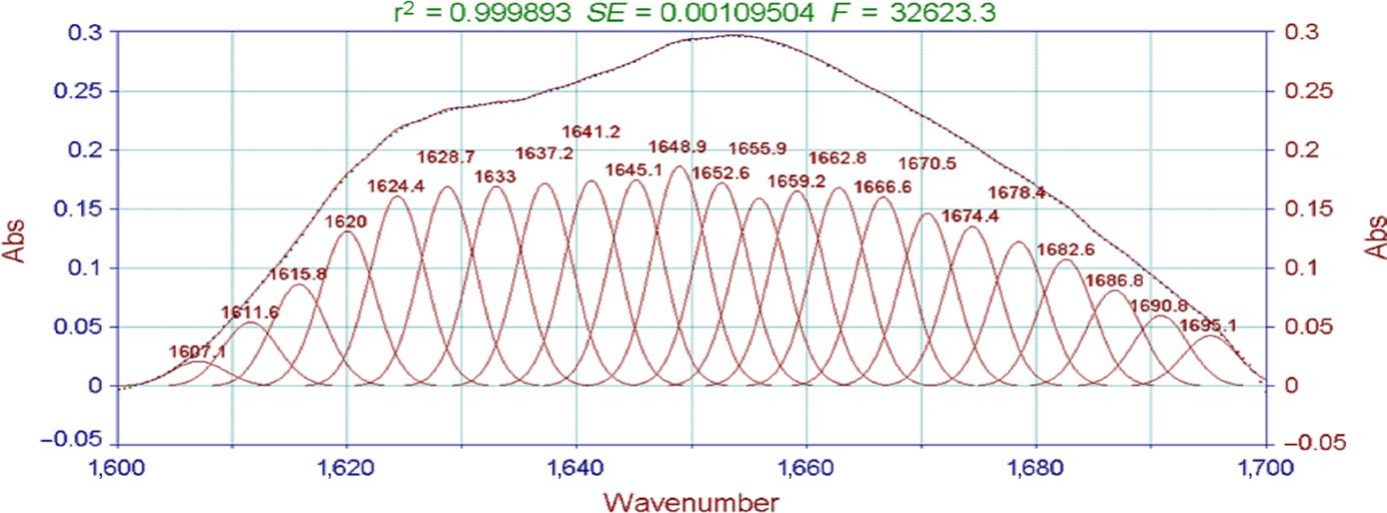
The fitting figures of amide I in surimi gel at 110°C

**Figure 11:**
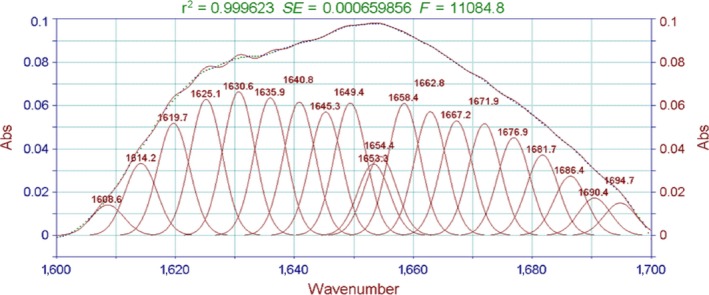
The fitting figures of amide I in surimi gel at 115°C

**Figure 12:**
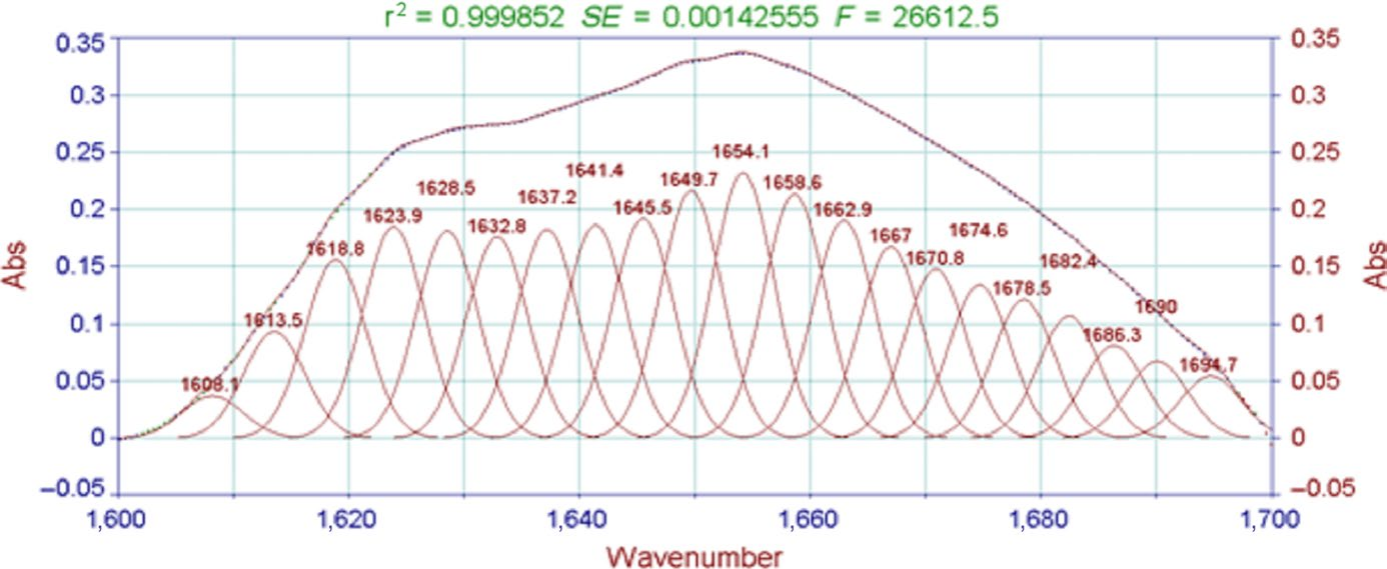
The fitting figures of amide I in surimi gel at 121°C

Moreover, the fitting figures of amide I in surimi gel at five different temperatures in Figures 8, 9, 10, 11, 12 and in Table 2 showed that surimi gel protein secondary structure is mainly β‐fold and β‐corner, and α‐helix content is relatively low. With the treating temperature increasing, β‐fold and random coil in surimi gel protein showed a significant decrease (*p* < 0.05). Especially, in the 110°C drops more sharply, suggesting that high temperature damaged the β‐fold and random coil structure. As the temperature continued to rise, secondary structure of protein was eventually destroyed, resulting in protein polymerization, which explains why the gel strength decreases as the treatment temperature increase.

**Table 2:** Change of secondary structure of surimi treated by different high‐temperatures

Temperature/℃	β‐fold	Random coil	α‐helix	β‐corner
100	34.69 ± 0.03^c^	20.35 ± 0.41^d^	15.25 ± 0.16^d^	35.31 ± 0.39^e^
105	36.80 ± 0.12^d^	20.45 ± 0.02^e^	13.84 ± 0.03^a^	32.51 ± 0.37^a^
110	31.25 ± 0.15^b^	17.71 ± 0.18^a^	16.45 ± 0.10^e^	33.89 ± 0.15^c^
115	32.48 ± 0.31^e^	16.85 ± 0.15^c^	14.41 ± 0.11^c^	33.48 ± 0.10^b^
120	31.21 ± 0.36^a^	17.07 ± 0.24^b^	14.27 ± 0.25^b^	34.24 ± 0.62^a^

The mean values marked with lowercase letters in ascending order show significant statistical differences within rows (p < 0.05).

### 3.6 E‐nose analysis of volatile compounds

**Figure 13:**
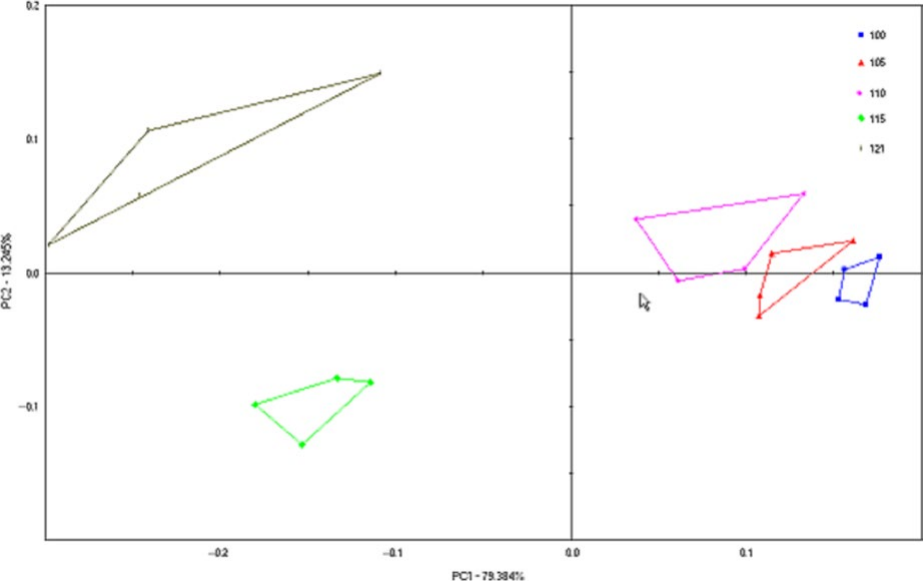
PCA plot of e‐nose response data for high‐temperature treatment of surimi gels

E‐nose analysis was carried out to evaluate the differences in the aroma profiles of five samples and a PCA plot and is shown in Figure 13. The plot consists of two axes, PC1 and PC2, in which PC1 explains 79.384% of the sample variance and PC2 explains only 13.245%. The total contribution rate is 92.629%. A total contribution rate of 75%–85% is considered an acceptable model explained most of the variance. The greater contribution rate beyond this range indicated that the main components can better reflect the original multi‐index information Lu et al. ([Link]). Therefore, the majority of the variations captured by PC1 for all the volatiles allows for the distinction between surimi samples submitted to different high‐temperature treatments. Although there were notably small variations, PC2 was still important in determining certain factors pertaining to the effects of the different temperature treatments.

Figure 13 shows that the dots corresponding to samples processed at 115 and 121°C are farther away from those corresponding to the samples processed at 100, 105, and 110°C, which are closest in the PCA, thus indicating the smallest difference in odor components among the three samples. The samples treated at 121°C were far from the other four groups in the PCA, indicating that they differed greatly in odor components. The results are consistent with those of the GC–MS analysis in 3.7, thus indicating that the higher the treatment temperature, the more obvious the effect on the volatile odor of the surimi gel.

### 3.7 Volatile compounds of Alaska pollock surimi gel

Alaska minced fish gel samples treated at 100, 105, 110, 115, and 120°C were detected 37, 46, 49, 52, and 56 volatile components, respectively. These were composed mainly of aldehydes, hydrocarbons, and a small number of aromatic substances, which is consistent with the finding of Cavalli's research (Cavalli, Fernandez, Lizzani‐Cuvelier, & Loiseau, 2003) that aldehydes are the major contributions of fish flavor formation.

With the increase in heating temperature, types of surimi gel volatile substances gradually increased correspondingly as shown in Table 3. While there were found to be some differences in composition and relative content, the major important flavor substances included hexanal, heptanal, nonanal, 1‐octen‐3‐ol, heptadecane, and 1‐penten‐3‐ol. Importantly, the 5‐methyl‐2‐heptene was only detected at 100°C, 6‐methyl‐2‐heptanone was detected in 110 and 115°C, while 1‐butanol was found only at 120°C treated samples. This suggests that as the temperature increases, numerous chemical changes occur in the surimi products such as lipid oxidation and Maillard reaction. In particular, this experiment detected additional compounds (toluene, benzene, naphthalene) that may not be originated from the change in surimi quality. Many of these additional compounds may indicate pollutants in the water that are transferred from the water to the fish (Rong, Qong, Zhang, Xie, & Xiong, 2015).

**Table 3:** Volatile components of Alaska pollock surimi gel extracted at different high temperature treatments

Category	Retenti‐on	Compounds	Time /min	Relative amount %				
				100°C	105°C	110°C	115°C	121°C
Aldehydes	2.617	Butanal, 3‐methyl‐	ND	—	1.16 ± 0.02	0.23 ± 0.02	0.14 ± 0.01	0.17 ± 0.03
	2.748	Pentanal	ND	0.29 ± 0.01	—	—	—	—
	4.288	2‐Pentenal, (E)‐	ND	—	—	—	—	1.31 ± 0.02
	4.482	Hexanal	4.5	8.12 ± 0.25^c^	9.67 ± 0.08^e^	8.98 ± 0.15^d^	5.56 ± 0.18^b^	5.01 ± 0.07^a^
	7.768	Heptanal	3	3.91 ± 0.12^e^	3.51 ± 0.02^d^	3.05 ± 0.08^c^	1.92 ± 0.03^a^	2.49 ± 0.08^b^
	8.213	4‐Heptenal, (E)‐	ND	—	3.47 ± 0.02	—	1.37 ± 0.06	—
	8.31	4‐Heptenal, (Z)‐	ND	2.66 ± 0.04	—	2.96 ± 0.31	—	—
	12.001	Octanal	ND	—	6.64 ± 0.23	6.20 ± 0.12	4.68 ± 0.10	—
	12.266	2,4‐Decadienal	0.07	—	—	—	0.40 ± 0.01	0.50 ± 0.04
	12.373	2,4‐Heptadienal, (E,E)‐	ND	0.69 ± 0.01^c^	0.48 ± 0.02^a^	0.51 ± 0.02^b^	0.99 ± 0.02^e^	0.93 ± 0.03^d^
	13.437	Benzaldehyde, 2‐hydroxy‐	ND	—	—	—	0.13 ± 0.01	0.38 ± 0.02
	14.124	2‐Octenal, (E)‐	3	0.7 ± 0.02^c^	0.45 ± 0.03^a^	—	0.59 ± 0.01^b^	0.75 ± 0.02^d^
	15.818	Nonanal	1	6.2 ± 0.11^d^	5.63 ± 0.20^b^	6.17 ± 0.15^d^	4.39 ± 0.08^a^	5.73 ± 0.13^c^
	17.614	2,6‐Nonadienal, (E,E)‐	ND	0.86 ± 0.01	—	—	—	0.72 ± 0.04
	18.485	Decanal	2	1.02 ± 0.01	1.08 ± 0.02	1.06 ± 0.02	0.79 ± 0.02	0.91 ± 0.01
	36.96	Tetradecanal	ND	0.31 ± 0.01	4.21 ± 0.06	2.51 ± 0.06	—	—
		Subtotal	ND	24.76	36.30	31.67	21.58	18.52
Ketone	7.956	2‐Heptanone	ND	0.54 ± 0.01	0.73 ± 0.01	0.59 ± 0.02	0.53 ± 0.02	—
	10.273	2‐Heptanone, 6‐methyl‐	ND	—	—	0.12 ± 0.01	0.12 ± 0.01	—
	10.665	2,3‐Octanedione	ND	4.09 ± 0.06	—	—	—	—
	11.452	3‐Octanone	ND	3.10 ± 0.05	1.57 ± 0.01	—	—	—
	11.646	2‐Octanone	ND	—	—	0.68 ± 0.01	0.54 ± 0.08	0.62 ± 0.01
	11.503	5‐Hepten‐2‐one, 6‐methyl‐	ND	—	—	—	—	0.69 ± 0.03
	15.423	3,5‐Octadien‐2‐one	ND	—	—	—	2.48 ± 0.05	—
	22.532	2‐Undecanone	ND	2.41 ± 0.02^e^	1.94 ± 0.05^d^	1.51 ± 0.04^b^	1.12 ± 0.03^a^	1.67 ± 0.07^c^
		Subtotal	ND	10.14	4.24	2.90	4.79	2.98
	2.508	1‐Butanol	ND	1.33 ± 0.05	1.36 ± 0.02	1.29 ± 0.04	0.99 ± 0.04	—
	2.943	1‐Penten‐3‐ol	ND	2.31 ± 0.07^b^	2.57 ± 0.12^c^	2.82 ± 0.06^d^	2.40 ± 0.14^b^	2.11 ± 0.05^a^
	4.557	1‐Pentanol	ND	—	—	—	0.44 ± 0.02	0.49 ± 0.03
Alcohols	4.643	2‐Penten‐1‐ol, (Z)‐	ND	0.65 ± 0.02^e^	0.45 ± 0.01^d^	0.44 ± 0.05^c^	0.36 ± 0.02^a^	0.41 ± 0.07^b^
	4.774	1‐Hexen‐3‐ol	ND	—	1.20 ± 0.02^c^	1.29 ± 0.03^d^	0.18 ± 0.02^a^	0.20 ± 0.01^b^
	7.258	1‐Hexanol	250	0.97 ± 0.02	—	—	1.26 ± 0.02	—
	10.834	1‐Heptanol	ND	1.57 ± 0.02	—	—	1.43 ± 0.01	—
	11.177	1‐Octen‐3‐ol	ND	17.31 ± 1.24^e^	13.61 ± 0.42^c^	14.47 ± 0.26^d^	7.22 ± 0.14a	12.35 ± 0.16^b^
	13.037	1‐Hexanol, 2‐ethyl‐	ND	2.7 ± 0.04^d^	3.89 ± 0.04^e^	1.75 ± 0.04^a^	2.10 ± 0.02^b^	2.50 ± 0.12^c^
	13.529	4‐Ethylcyclohexanol	ND	—	—	1.15 ± 0.01	0.97 ± 0.04	—
	13.693	2‐Octen‐1‐ol, (Z)‐	40	—	—	—	1.94 ± 0.11	1.48 ± 0.06
	18.885	5‐Decanol	ND	0.27 ± 0.01^d^	0.37 ± 0.08e	0.26 ± 0.03^c^	0.20 ± 0.02^a^	0.25 ± 0.01^b^
	21.803	1‐Decanol	ND	—	—	0.72 ± 0.03	—	1.21 ± 0.05
	29.147	1‐Decanol, 2‐hexyl‐	ND	—	—	—	0.44 ± 0.03	0.56 ± 0.03
	32.142	Cedrol	ND	2.97 ± 0.23^a^	5.32 ± 0.12^c^	5.53 ± 0.19^d^	4.26 ± 0.07^b^	12.28 ± 0.49^e^
		Subtotal		30.08	28.74	29.63	23.57	32.36
Hydrocarbo‐ns	2.102	n‐Hexane	ND	—	0.59 ± 0.02^b^	0.91 ± 0.06^d^	0.54 ± 0.04^a^	0.76 ± 0.10^c^
	5.003	3‐Octyne	ND	0.54 ± 0.01	—	—	—	—
	5.186	Octane	ND	—	—	—	—	0.48 ± 0.01
	5.351	2‐Heptene, 5‐methyl‐	ND	1.47 ± 0.02	—	—	—	—
	5.381	3‐Octene, (E)‐	ND	—	0.68 ± 0.03	—	—	—
	5.438	2,5‐Octadiene	ND	—	2.57 ± 0.04	2.86 ± 0.03	2.01 ± 0.03	—
	10.839	1‐Hexene, 3,5,5‐trimethyl‐	ND	—	—	—	1.15 ± 0.02	1.49 ± 0.06
	11.841	Cyclohexene	ND	0.47 ± 0.01	—	—	—	—
	12.928	D‐Limonene	ND	—	0.55 ± 0.18^d^	0.42 ± 0.02^b^	0.39 ± 0.04^a^	0.50 ± 0.05^c^
	14.604	1,3‐Cyclooctadiene	ND	13.16 ± 0.47^d^	9.52 ± 0.10^c^	1.28 ± 0.11^a^	7.60 ± 0.21^b^	—
	15.148	1‐(2‐Propenyl)cyclopentene	ND	—	2.36 ± 0.01	1.81 ± 0.02	—	—
	15.634	Undecane	ND	0.43 ± 0.01^b^	0.49 ± 0.06^c^	0.56 ± 0.01^e^	0.37 ± 0.01^a^	0.51 ± 0.02^d^
	19.256	Dodecane	ND	0.4 ± 0.01^b^	0.50 ± 0.03^d^	0.44 ± 0.01^c^	0.29 ± 0.04^a^	0.45 ± 0.01^c^
	21.654	4‐Octyne	ND	—	1.47 ± 0.04	2.00 ± 0.01	—	—
	22.707	Tridecane	ND	—	0.26 ± 0.01	0.40 ± 0.01	—	0.27 ± 0.01
	22.985	Tetradecane	ND	1.4 ± 0.03^e^	0.85 ± 0.11^a^	1.09 ± 0.02^c^	0.94 ± 0.02^b^	1.23 ± 0.01^d^
	29.081	Pentadecane	ND	2.21 ± 0.04^c^	1.48 ± 0.02^a^	2.43 ± 0.03^d^	1.85 ± 0.05^b^	2.42 ± 0.06^d^
	32.394	Oxirane, decyl‐	ND	—	—	—	—	2.11 ± 0.10
	37.578	Hexadecane	ND	—	—	—	—	1.85 ± 0.02
	34.774	Heptadecane	ND	6.43 ± 0.10	3.54 ± 0.06	4.17 ± 0.01	6.01 ± 0.10	8.99 ± 0.21
	36.680	Octadecane	ND	0.45 ± 0.04	1.48 ± 0.01	1.23 ± 0.04	3.42 ± 0.02	6.68 ± 0.08
	36.960	Oxirane, hexadecyl‐	ND	—	—	—	2.64 ± 0.04	3.32 ± 0.02
Aromatic compound	35.947	1‐Nonadecene	ND	—	—	—	1.80 ± 0.03	—
	38.167	Nonadecane	ND	0.37 ± 0.02	—	—	—	—
	39.421	Eicosane	ND	—	0.56 ± 0.03	0.42 ± 0.03	5.12 ± 0.01	0.56 ± 0.20
	23.931	Hexacosane	ND	—	—	0.35 ± 0.02	—	—
	31.170	Tetratetracontane	ND	—	—	0.15 ± 0.01	—	—
		Subtotal		27.33	26.90	20.50	34.13	31.62
	1.684	Methylamine, N,N‐dimethyl‐	ND	—	3.92 ± 0.04	2.50 ± 0.11	2.20 ± 0.81	0.72 ± 0.01
	3.201	Furan, 2‐ethyl‐	ND	—	2.06 ± 0.03	1.80 ± 0.09	—	2.81 ± 0.12
	4.419	Toluene	ND	—	0.33 ± 0.02	0.32 ± 0.05	—	0.25 ± 0.02
	7.229	p‐Xylene	ND	—	—	—	—	0.20 ± 0.01
	7.927	Styrene	ND	—	—	—	—	0.56 ± 0.04
	8.98	Phenol, 4‐ethyl‐	ND	—	0.70 ± 0.01^d^	0.37 ± 0.02^b^	0.31 ± 0.01^a^	0.65 ± 0.02^c^
	10.416	Benzaldehyde	ND	5.22 ± 0.05	—	—	—	3.17 ± 0.10
	11.251	Benzene	ND	0.67 ± 0.01	1.05 ± 0.01	0.12 ± 0.01	0.09 ± 0.01	0.13 ± 0.02
	11.217	Phenol	ND	—	—	—	0.70 ± 0.06	—
	11.589	Furan, 2‐pentyl‐	ND	—	—	1.26 ± 0.03	1.05 ± 0.01	1.99 ± 0.13
	14.364	Acetophenone	ND	—	—	—	—	0.59 ± 0.04
	17.368	Benzene, 1,2‐dimethoxy‐	ND	—	—	—	—	—
	17.912	Benzaldehyde, 4‐ethyl‐	ND	—	2.05 ± 0.05	1.17 ± 0.01	—	1.46 ± 0.03
	18.564	Naphthalene	60	0.74 ± 0.03	0.71 ± 0.01	—	—	0.56 ± 0.01
	22.438	Naphthalene, 2‐methyl‐	ND	—	—	—	1.11 ± 0.02	1.51 ± 0.02
	22.981	Naphthalene, 1‐methyl‐	ND	1.15 ± 0.01	1.00 ± 0.01	0.71 ± 0.06	—	0.53 ± 0.07
	26.066	Naphthalene, 1,6‐dimethyl‐	ND	—	0.46 ± 0.09	0.39 ± 0.04	—	—
	29.47	Phenol, 2,4‐bis(1,1‐dimethylethyl)‐	ND	—	0.53 ± 0.01^b^	0.30 ± 0.01^a^	0.55 ± 0.02^c^	0.74 ± 0.07^d^
	41.263	Decanedioic acid, dibutyl ester	ND	—	—	—	0.27 ± 0.02	0.34 ± 0.01
		Subtotal		8.08	12.81	8.94	6.28	16.21

The mean values marked with lowercase letters in ascending order show significant statistical differences within rows (p < 0.05).

## 4 CONCLUSION

The quality and structure strength of the surimi gel and volatile component are changed in varying degrees as after the heat treatment temperature increases. The gel strength is the most important quality indicator for surimi products. High‐temperature treatment damages surimi gel properties to some extent. The result of FT‐IR spectroscopy in this study suggests that with increase in temperature, protein secondary structure of the random coil received maximum damage, leading to protein aggregation and ultimately greatly reduced gel strength. Further research is thus required into the benefits of alternative and more advanced methods for preservation of fish and surimi products.
